# Neural reconstruction of bone-eating *Osedax* spp. (Annelida) and evolution of the siboglinid nervous system

**DOI:** 10.1186/s12862-016-0639-7

**Published:** 2016-04-14

**Authors:** Katrine Worsaae, Nadezhda N. Rimskaya-Korsakova, Greg W. Rouse

**Affiliations:** Marine Biological Section, Department of Biology, University of Copenhagen, Universitetsparken 4, DK-2100 Copenhagen, Denmark; Department of Invertebrate Zoology, Lomonosov Moscow State University, Leninskie Gory 1-12, Moscow, 119991 Russia; Scripps Institution of Oceanography, 9500 Gilman Drive, La Jolla, California 92093-0202 USA

**Keywords:** Cephalic ganglion, Ventral nerve cord, Neurophylogeny, Segmentation, Immunocytochemistry, Palp, Acetylated α-tubulin, Serotonin, FRMFamide, Immunoreactivity, CLSM, TEM, Histology

## Abstract

**Background:**

Bone-devouring *Osedax* worms were described over a decade ago from deep-sea whale falls. The gutless females (and in one species also the males) have a unique root system that penetrates the bone and nourishes them via endosymbiotic bacteria. Emerging from the bone is a cylindrical trunk, which is enclosed in a transparent tube, that generally gives rise to a plume of four palps (or tentacles). In most *Osedax* species, dwarf males gather in harems along the female’s trunk and the nervous system of these microscopic forms has been described in detail. Here, the nervous system of bone-eating *Osedax* forms are described for the first time, allowing for hypotheses on how the abberant ventral brain and nervous system of Siboglinidae may have evolved from a ganglionated nervous system with a dorsal brain, as seen in most extant annelids.

**Results:**

The intraepidermal nervous systems of four female *Osedax* spp. and the bone-eating *O. priapus* male were reconstructed in detail by a combination of immunocytochemistry, CLSM, histology and TEM. They all showed a simple nervous system composed of an anterior ventral brain, connected with anteriorly directed paired palp and gonoduct nerves, and four main pairs of posteriorly directed longitudinal nerves (2 ventral, 2 ventrolateral, 2 sets of dorso-lateral, 2 dorsal). Transverse peripheral nerves surround the trunk, ovisac and root system. The nervous system of *Osedax* resembles that of other siboglinids, though possibly presenting additional lateral and dorsal longitudinal nerves. It differs from most Sedentaria in the presence of an intraepidermal ventral brain, rather than a subepidermal dorsal brain, and by having an intraepidermal nerve cord with several plexi and up to three main commissures along the elongated trunk, which may comprise two indistinct segments.

**Conclusions:**

*Osedax* shows closer neuroarchitectural resemblance to Vestimentifera + *Sclerolinum* (= Monilifera) than to Frenulata. The intraepidermal nervous system with widely separated nerve cords, double brain commissures, double palp nerves and other traits found in *Osedax* can all be traced to represent ancestral states of Siboglinidae. A broader comparison of the nervous system and body regions across *Osedax* and other siboglinids allows for a reinterpretation of the anterior body region in the group.

## Background

Siboglinidae is a group of annelids including Frenulata, Vestimentifera and *Sclerolinum* (the latter two referred to here as Monilifera, see Rouse 2001 [1]) that can be found at deep-sea reduced environments such as hydrothermal vents and cold-water sulfide/hydrocarbon seeps [2–4]. One of the most recently named Siboglinidae genera is *Osedax,* described from vertebrate bones [5, 6]. The females possess an elongated trunk with four anterior appendages (termed palps or tentacles, here referred to as palps) and a unique posterior branching structure, a so-called root system, which penetrates the bones and house bacteriocytes [6]. All siboglinids lack a mouth and digestive tract as adults and are dependent on bacterial endosymbionts [7–9]. The slender long body of frenulates is less than one mm wide but more than 50 cm long, while species such as the giant tubeworm, *Riftia* (Vestimentifera), can reach a length of over a meter [10, 11], whereas the *Osedax* females are maximally 15 cm long and their dwarf males usually less than one mm in length [6, 12].

The phylogenetic position of Siboglinidae within Annelida is not yet settled [13–19] and various sister groups have been proposed or recovered, e.g., Oweniidae [15, 17, 20, 21], Sabellida [13, 19], Cirratuliformia [22, 23], or Clitellata [18, 24]. Within Siboglinidae, *Osedax* was initially found to be sister group to Monilifera (Vestimentifera + *Sclerolinum*) [6, 25–27]. Some recent analyses [28, 29] have placed *Osedax* as closer to Frenulata, but the placement with Monilifera now seems well supported [27]*.*

The siboglinid body regions have been described with varying terminology, interpretations and homology arguments [1, 2, 30, 31]. Based on development in vestimentiferans, Bright et al. [31] reconciled the various siboglinid anatomical terms with those of annelids as follows: 1) the ‘cephalic lobe + forepart’ of frenulates and *Sclerolinum*, and the ‘vestimentum’ of Vestimentifera consist of the merged prostomium, peristomium and the anterior end of the first chaetiger; 2) the ‘trunk’ of Vestimentifera, *Sclerolinum* and Frenulates consist of the posterior part of the first chaetiger; and 3) the ‘opisthosoma’ represents the remaining segments (inclusive the last segment = pygidium). *Osedax* was described with annelid terminology [6], though no explicit reference to a head was made beyond describing a crown with an oviduct and four pinnule-bearing palps in females. Later descriptions of both dwarf males [12, 28], and bone-eating males and females [29] mention a prostomium. The palps of *Osedax japonicus* were suggested to be of prostomial origin [32], though the exact border of the prostomium and peristomium is not yet established in *Osedax* and more detailed developmental studies are necessary to confirm their affinity. The palp or palps (or tentacle/tentacles) of Frenulata have been regarded to be either prostomial [31], or peristomial [1] based on their origin posterior to the prototroch. Likewise, the vestimentiferan branchial plume has been suggested to be either prostomial [33], or peristomial [1, 13], or with affinity to the first chaetiger [31]. Regardless the origin and terminology, all siboglinid palps (or tentacles or plumes) have been regarded as homologous structures [1], though detailed studies on their innervation and nervous connection to the brain is lacking. The forepart of frenulates and *Sclerolinum* and the vestimentum of Vestimentifera has been proposed homologous to the entire (or at least anterior) *Osedax* trunk [6, 12, 32, 34, 35]. The posterior root system of *Osedax* (as well as the internal ovisac) has been proposed homologous to the posterior elongated ‘trunk’ (containing the trophosome) of other siboglinids that likewise contains the bacterial endosymbionts [30, 32, 36]. The posteriormost opisthosoma found in most Siboglinidae is missing or strongly reduced in bone-eating *Osedax* specimens [6]. In *Osedax* larvae and dwarf males the two successive dorsal and ventral pairs of posterior opisthosomal chaetae arguably reflect the presence, at least during development, of an opisthosomal region homologous to that of other Siboglinidae [6, 12, 37]. A better understanding of the internal organ systems such as the nervous system may aid in solving homology and terminology issues with regard to siboglinid segments and body regions.

Monilifera and Frenulata possess a similar general outline of their intraepidermal nervous system consisting of a ventral brain, which posteriorly extends into a paired medio-ventral cord, lacking segmental ganglia, except in the opisthosoma [10, 11, 26, 38, 39]. This stands in contrast to the subepidermal nervous system of most Sedentaria, consisting of a dorsal brain connected through the circumesophageal connectives to 1-5 ventral cords, containing a subesophageal ganglion followed by multiple segmental paired ganglia [40, 41]. Unsurprisingly, with the lack of a mouth, gut or obvious trunk segmentation in adult siboglinids, no circumesophageal connectives and stomatogastric nervous system, nor segmental trunk ganglia are found in adults. Furthermore, the nervous system has only been investigated in detail for a few species, mainly based on classic histology and light microscopy studies.

The vestimentiferan nervous system shows following characteristics: a ventral cord originating as a single median cord at the posterior edge of the brain and extending intraepidermally along the midline of the trunk and opisthosoma to the posteriormost end. The cord also contains a pair of giant axons, accommodating fast contraction into the tube. Beneath the ventral ciliated field of the vestimentum, the cord briefly separates into two strands forming a dense plexus of tiny nerves, innervating the ciliary field. In each segment of the opisthosoma, the median cord gives rise to a pair of segmental ring nerves [11, 31, 42–47]. The brain of juvenile Vestimentifera was found to originate dorsally above the transient mouth opening, although the brain of all adult siboglinids is positioned antero-ventrally [48, 49]. The large adult brain shows a central neuropil surrounded by dense ventral as well as dorsal layers of perikarya [11, 31, 43, 44, 46, 47]. The neurites of the dorsal perikarya form the plume nerve bundles, extending anteriorly to the plume lamellae [2, 45]. The cuticle forms a thick shield ventrally, presumably hereby protecting the ventral, intraepidermally positioned brain [45]. A medio-dorsal nerve cord has only been described for *Lamellibrachia satsuma* Miura, Tsukahara, Hashimoto, 1997 [47, 50], but may be found in other species following similar thorough examinations. There is little knowledge on the receptor cells and peripheral nervous system, which is complicated by the large size of the Vestimentifera. *Sclerolinum*, the sister group to Vestimentifera within Monilifera*,* has a similar nervous system, with one medio-ventral cord, producing a wide nerve net underneath the midventral ciliary field along the forepart [26, 51]. Frenulata likewise has a medio-ventral nerve cord extending from the ring-shaped brain commissure in the cephalic lobe to the segmented opisthosoma, where three-segmented ganglia has been described for *Siboglinum fiordicum* Webb, 1963 [52, 53], possibly innervating the segmental muscles moving the chaetae. The small frenulate *Nereilinum murmanicum* Ivanov, 1961 [54] differs from other siboglinids in having separate, paired ventral cords along the entire forepart as well as trunk; the nerve net of which innervates the long locomotory mid-ventral ciliary band used for gliding [55].

The nervous system of the fourth major siboglinid clade, *Osedax*, has been described for the paedomorphic dwarf males, which have an anterior brain with two commissures, a ring nerve underlying the prototroch and three pairs of ventral and lateral nerves extending along the trunk interconnected by two posterior transverse commissures [12]. Little is known about the female nervous system [34]. The microscopic dwarf males were shown to have an unusual, though very simple nervous system that is not easily compared to other siboglinids or annelid nervous systems [12]. However, the recent description of the secondarily reversed, large males of *Osedax priapus* Rouse, Wilson, Worsaae, Vrijenhoek, 2015 [29] also gave a glimpse of a more complex nervous system, including a ventral brain and paired ventral cords (suppl. figure S6H-K, [29]). In the description of female *Osedax roseus* Rouse, Worsaae, Johnson, Jones, Vrijenhoek, 2008 [56], the remarkably densely stained serotonergic dorsal nerves were mistakenly taken for ventral cords, which led to a misinterpretation of the dorso-ventral orientation [34]. The correct dorso-ventral axis was established in a following paper [57] examining the blood vascular system and identifying the forward pumping of blood by a muscular dorsal vessel [57–59]; an orientation later supported by anatomical details on cilia, nerve cords, brain and oviducts matching that of other siboglinids [29].

With the present paper we aim to describe the adult nervous system of bone-eating *Osedax* forms*,* covering the possible variation across four species, visualized with immunoreactivity (IR) of the cytoskeleton and two neurotransmitters gathered with confocal laser scanning microscopy. Complimentary data from TEM and traditional histological LM microscopy are also added. The results are compared with previous data on other siboglinids and the evolution of nervous system within Siboglinidae is discussed. Furthermore, the homology and segmentation of the different body regions in Siboglinidae is interpreted relative to the configuration of their nervous system.

## Results

### General composition of nervous system

**Fig. 1:**
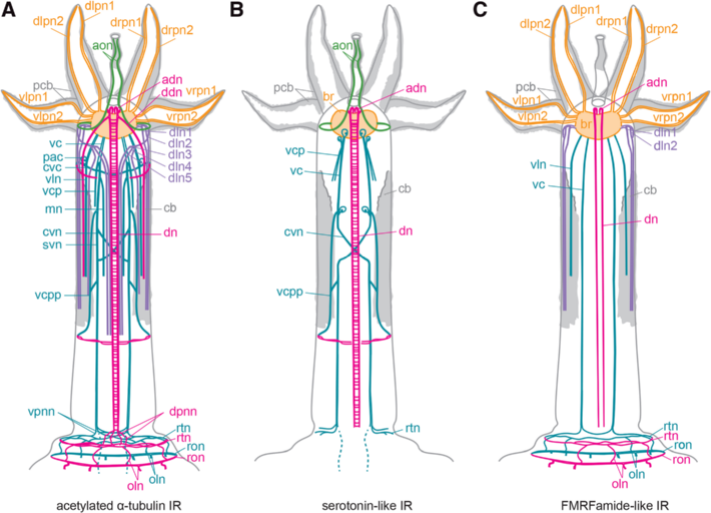
Schematic drawings of female *Osedax mucofloris* nervous system, based upon immunohistochemical studies. **a** Drawing of the nervous system visualized with acetylated α-tubulin IR in CLSM (entire nervous cytoskeleton). **b** Drawing of the serotonin LIR. **c** Drawing of the FRMFamide LIR in female *Osedax* nervous system*.* Color codes: blue, ventral nerves; green, oviduct nerves; grey, body outline; orange, palp nerves; pink, dorsal nerves; purple, dorso-lateral nerves; white, ciliary bands. Abbreviations: *adn* – antero-dorsal nerve, *aon* – anterior oviduct nerve, *br* – brain, *cb* – ciliary band, *cvc* – commissure of the *vc*, *cvn* – crossing neurites of the *vc*, *ddn* – dorso-diagonal nerve, *dn* – dorsal nerve, *dln1-5* – dorso-lateral nerves 1-5, *dlpn1,2* – dorsal left palp nerve 1, 2, *dpnn* – dorsal posterior nerve net, *drpn1, 2* – dorsal right palp nerve 1, 2, *mn* – median nerve, *oln* – ovisac longitudinal nerves, *pac* – perikarya of the *cvc*, *pcb* – palp ciliary band, *ron* – ring ovisac nerve, *rtn* – ring nerve at the base of the trunk, *svn* – straight ventral neurites of the *vc, vc* – ventral cord, *vcp* – ventral cord projection, *vcpp* – posterior process of the *vc*, *vln* – ventro-lateral nerve, *vlpn1, 2* – ventral left palp nerve 1, 2, *vrpn1, 2* - ventral right palp nerve 1, 2

**Fig. 2:**
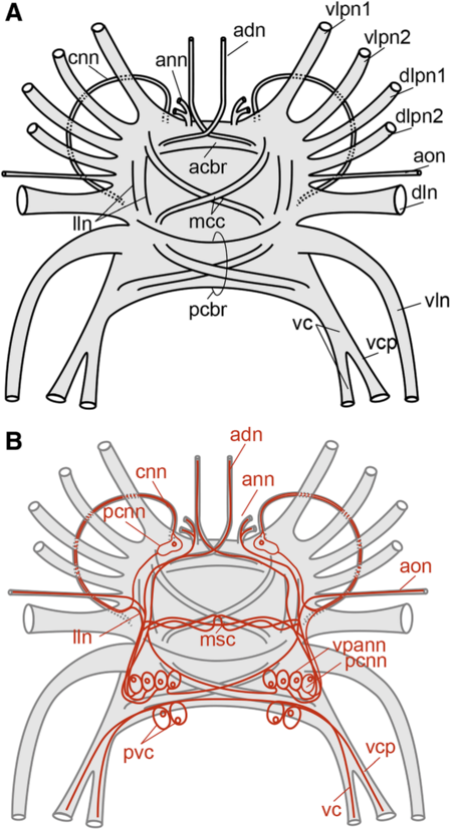
Schematic drawing of female *Osedax* “nudepalp E” brain, based upon immunohistochemical studies. Acetylated α-tubulin IR shown in black, serotonin LIR shown in red. Only major neurite bundles shown, minor bundles and neurites traversing the neuropil are not shown for clarity. Abbreviations: *acbr* – anterior commissure of the brain, *adn* – antero-dorsal nerve, *ann* – anterior nerve net, *aon* – anterior oviduct nerve, *cnn* – circular nerve net, *dln* – dorso-lateral nerve bundle, *dlpn 1,2* – dorsal left palp nerve 1, 2, *lln* – lateral longitudinal neurite bundles in the brain, *mcc* – middle cross commissure, *msc* – middle serotonergic commissure, *pcbr* – posterior commissure of the brain, *pcnn* – perikarya of the *cnn*, *pvc* – perikarya of the *vc*, *vc* – ventral cord, *vcp* – ventral cord projection, *vln* – ventro-lateral nerve, *vlpn1,2* – ventral left palp nerve 1,2, *vpann* – ventral perikarya of *ann*

**Fig. 3:**
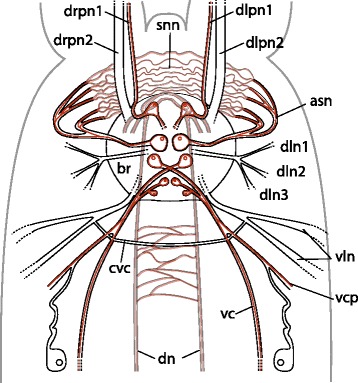
Schematic drawing of the nervous system of the anterior part of bone-eating male *Osedax priapus*. Acetylated α-tubulin IR shown in black, serotonin LIR shown in red, body outline in grey. Abbreviations: *asn* – anterior seminal nerve, *br* – brain, *cvc* – commissure of the *vc*, *dn* – dorsal nerve, *dln 1-3* – dorso-lateral nerve bundle 1-3, *dlpn1,2* – dorsal left palp nerve 1, 2, *drpn 1, 2* – dorsal right palp nerve 1, 2, *snn* – seminal vesicle nerve net, *vc* – ventral cord, *vcp* – ventral cord projection, *vln* – ventro-lateral nerve

**Fig. 4:**
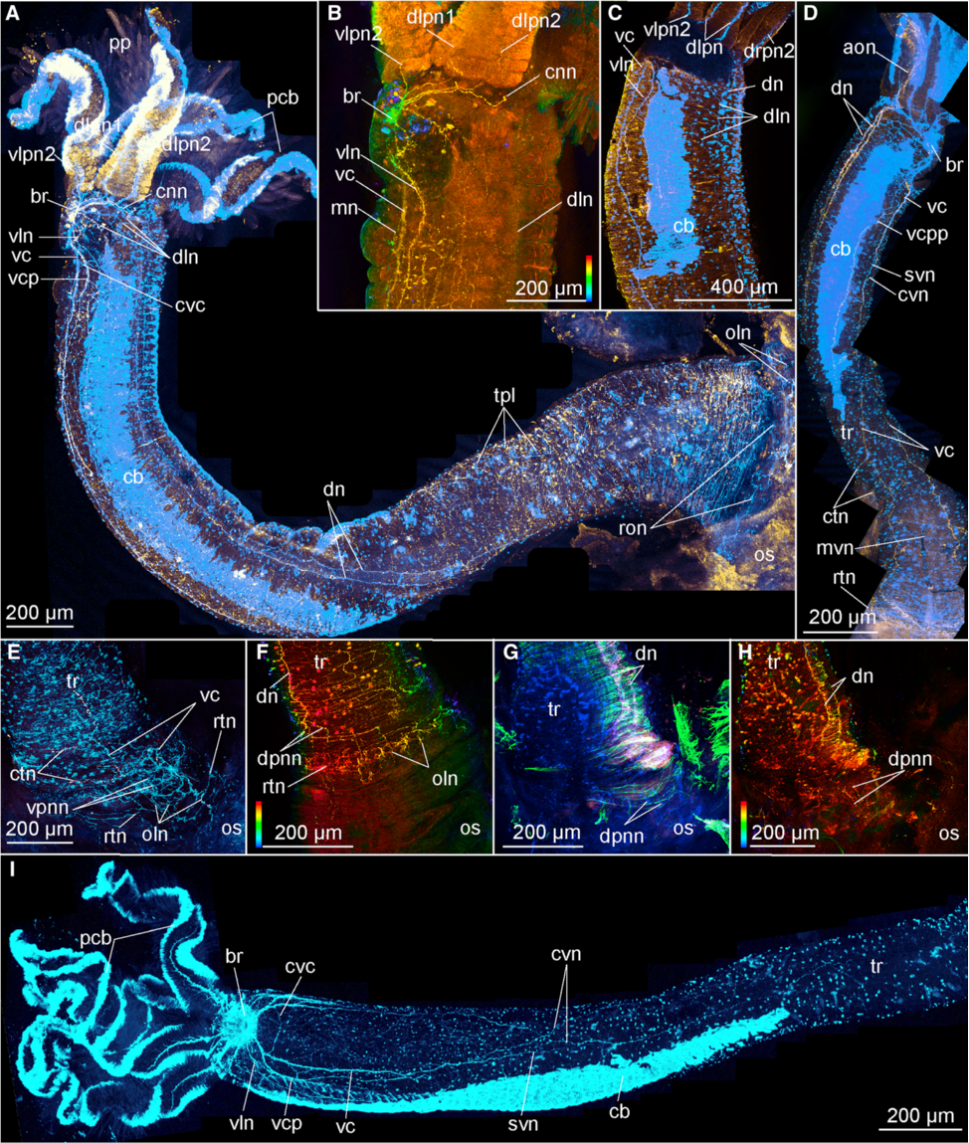
Confocal laser scanning microscopy of female *Osedax mucofloris (* **a** *,* **b** *,* **e** *-* **i** *) and O.* yellow collar (**c**, **d**) Depth coded images (in **b**, **f**, **h**) indicate the depth of the signal in the Z-stack: scale follows the colors of the spectral light, the top is to the surface. **a** Whole specimen (red, FMRFamide LIR; blue and cyan, acetylated α-tubulin IR), darker patch posteriorly to brain is an artifact of the labelling procedure. **b** Lateral view of anterior region (depth coded colors, FMRFamide LIR). **c** Lateral view of anterior region of trunk (red, FMRFamide LIR; blue, anti α-tubulin IR). **d** Whole specimen (red, anti serotonin LIR; blue, anti acetylated α-tubulin IR). **e** Ventral side of the posterior trunk (pink, serotonin LIR; blue, acetylated α-tubulin IR). **f** Lateral view of the posterior trunk (depth coded colors, acetylated α-tubulin IR). **g** Dorsal view of the posterior trunk (pink, serotonin LIR; blue, acetylated α-tubulin IR; green, phalloidin). **h** Dorsal view of the posterior trunk (depth coded colors, acetylated α-tubulin IR). **i** Ventral view of the anterior trunk showing straight and crossing neurite bundles of the ventral cords. Abbreviations: *aon* – anterior oviduct nerve, *br* – brain, *cb* – ciliary band, *cnn* – circular nerve net, *ctn* – circular trunk neurites, *cvc* – commissure of the *vc*, *cvn* – crossing neurites of the *vc, dn* – dorsal nerve, *dln* – dorso-lateral nerve bundle, *dlpn1,2* – dorsal left palp nerve 1, 2, *dpnn* – dorsal posterior nerve net, *drpn 2* – dorsal right palp nerve 2, *mn* – median nerve, *mvn* - median ventral nerve, *oln* – ovisac longitudinal nerves, *os* – ovisac, *pcb* – palp ciliary band, *pp* – palp pinules, *ron* – ring ovisac nerve, *svn* – straight ventral neurites of the *vc, tpl* – trunk plexus, *tr* – trunk, *vc* – ventral cord, *vcp* – ventral cord projection, *vcpp* – posterior process of the *vc*, *vln* – ventro-lateral nerve, *vlp 2* – ventral left palp nerve 2, *vpnn –* ventral posterior nerve net

**Fig. 5:**
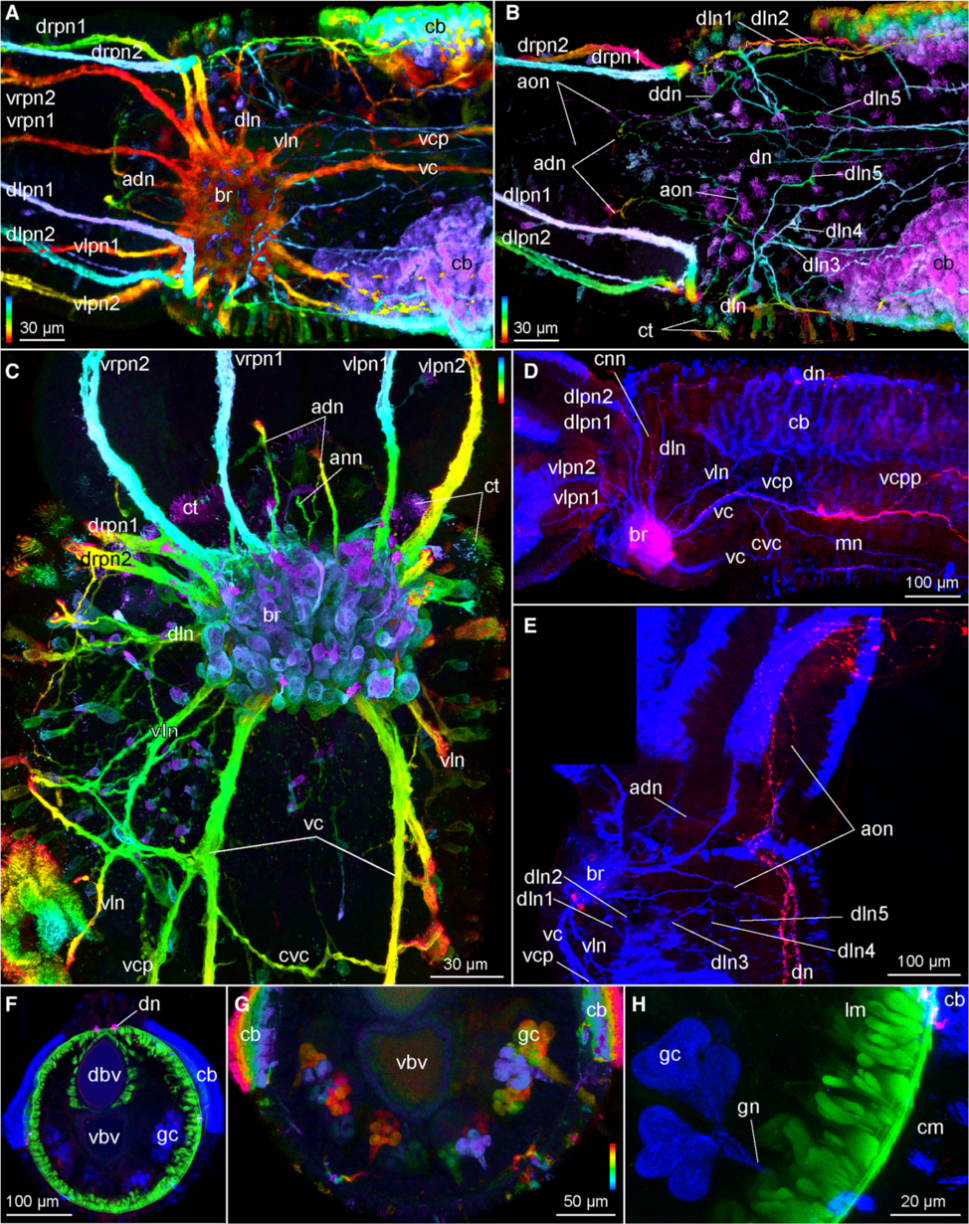
Confocal laser scanning microscopy of female *Osedax mucofloris*. Depth coded images (in **a**-**c**, **g**) indicate the depth of the signal in the Z-stack: scale follows the colors of the spectral light, the top is to the surface. **a** Dorsal view of anterior region, total depth of Z-stack (depth coded colors, acetylated α-tubulin IR). **b** Dorsal view of the dorsalmost part of Z-stack only (depth coded colors, acetylated α-tubulin IR). **c** Ventral view of the anterior region showing brain (depth coded colors, acetylated α-tubulin IR). **d** Ventro-lateral view of the anterior region, the lateralmost part of Z-stack only (red, serotonin LIR; blue, acetylated α-tubulin IR). **e** Lateral view of anterior region (red, serotonin LIR; blue, acetylated α-tubulin IR). The black box in the upper left corner is an artifact of the stiching of two images that are not overlapping in this area. **f** Cross section of trunk (green, phalloidin; blue, acetylated α-tubulin IR; red, serotonin LIR). **g** Close up of the ventral part of (**f**) showing glands (depth coded colors, acetylated α-tubulin IR). **h** Close up of glands from (**g**) (green, phalloidin; blue, acetylated α-tubulin IR; red, serotonin LIR). Abbreviations: *and* – antero-dorsal nerve, *ann*– anterior nerve net, *aon* – anterior oviduct nerve, *br* – brain, *cb* – ciliary band, *cm* – circular muscles, *cnn* – circular nerve net, *ct* – ciliary tuft, *cvc* – commissure of the *vc*, *dbv* – dorsal blood vessel, *ddn* – dorso-diagonal nerve, *dn* – dorsal nerve, *dln* – dorso-lateral nerve bundle, *dln1-5* – dorso-lateral nerves 1-5, *dlpn1,2* – dorsal left palp nerve 1, 2, *drpn1, 2* – dorsal right palp nerve 1, 2, *gc* – glandular cell, *gn* – glandular cell neck, *mn* – median nerve, *vbv* – ventral blood vessel, *vc* – ventral cord, *vcp* – ventral cord projection, *vcpp* – posterior process of the *vc*, *vln* – ventro-lateral nerve, *vlpn1, 2* – ventral left palp nerve 1, 2, *vrpn1, 2* - ventral right palp nerve 1, 2

**Fig. 6:**
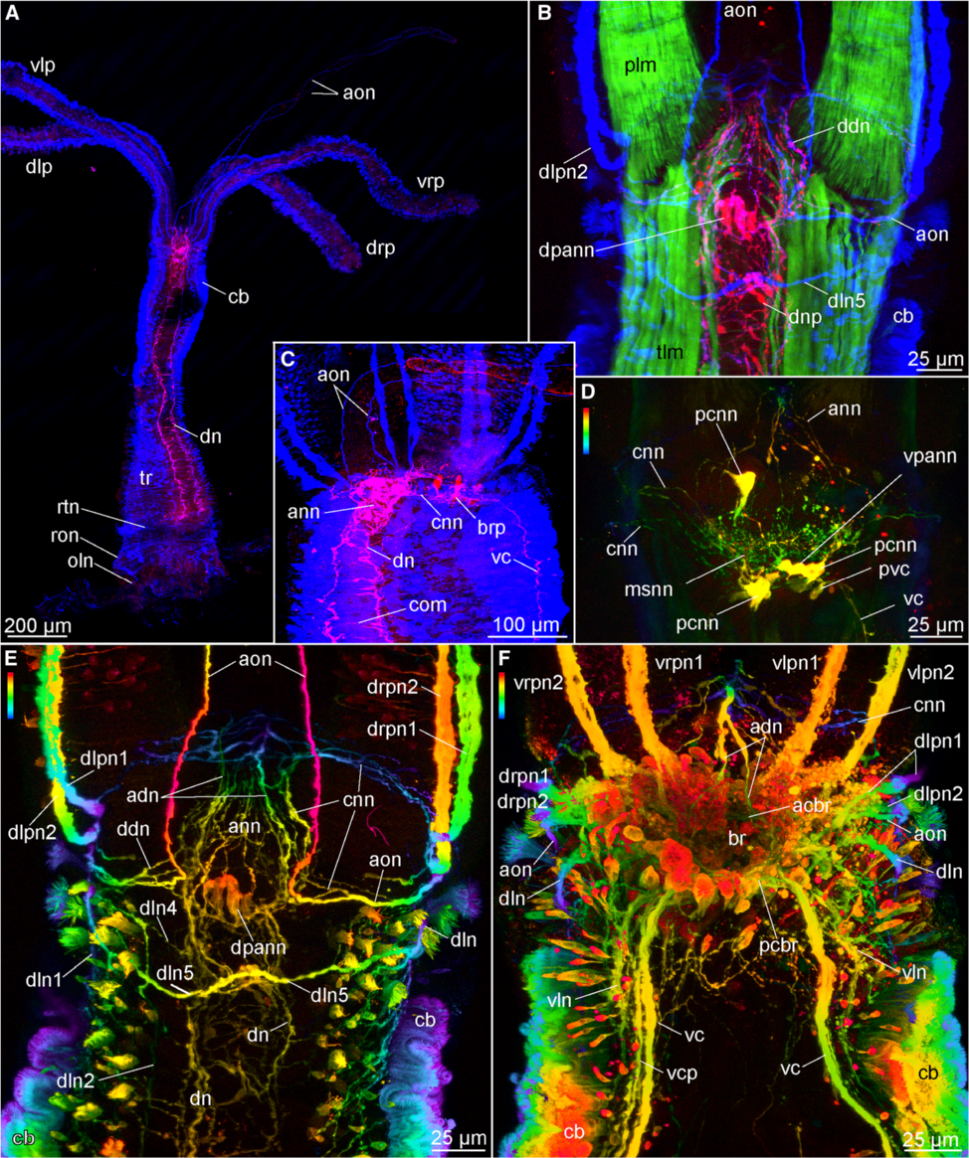
Confocal laser scanning microscopy of female *Osedax* “nudepalp E”. Depth coded images (in D-F) indicate the depth of the signal in the Z-stack: scale follows the colors of the spectral light, the top is to the surface. **a** Overview, dorsal view (blue, acetylated α-tubulin IR; red, serotonin LIR). **b** Anterior end, dorsal view (green, phalloidin; blue, acetylated α-tubulin IR; red, serotonin LIR). **c** Anterior end, lateral view (blue, acetylated α-tubulin IR; red, serotonin LIR). **d** Brain, ventral view (depth coded colors, serotonin LIR). **e** Anterior end, dorsal view (depth coded colors, acetylated α-tubulin IR). **f** Anterior end, ventral view (depth coded colors, acetylated α-tubulin IR). Abbreviations: *acbr* – anterior commissure of the brain, *adn* – antero-dorsal nerve, *ann* – anterior nerve net, *aon* – anterior oviduct nerve, *br* – brain, *brp* – brain perikarya, *cb* – ciliary band, *cnn* – circular nerve net, *com* – commissure, *ddn* – dorso-diagonal nerve, *dn* – dorsal nerve, *dln* – dorso-lateral nerve bundle, *dln1-5* – dorso-lateral nerves 1-6, *dlp* – dorsal left palp, *dlpn1,2* – dorsal left palp nerve 1, 2, *drp* – dorsal right palp, *drpn1, 2* – dorsal right palp nerve 1, 2, *msnn –* median serotonin-lir nerve net, *oln* – ovisac longitudinal nerves, *pcbr* – posterior commissure of the brain, *pcnn* – perikarya of the *cnn*, *pvc* – perikarya of the *vcrn* – root nerve, *ron* – ring ovisac nerve, *rtn* – ring nerve at the base of the trunk, *tr* – trunk, *vc* – ventral cord, *vcp* – ventral cord projection, *vln* – ventro-lateral nerve, *vlp* – ventral left palp, *vlpn1, 2* – ventral left palp nerve 1, 2, *vpann* – ventral perikarya of *ann*, *vrpn1, 2* - ventral right palp nerve 1, 2, *vrp* – ventral right palp

**Fig. 7:**
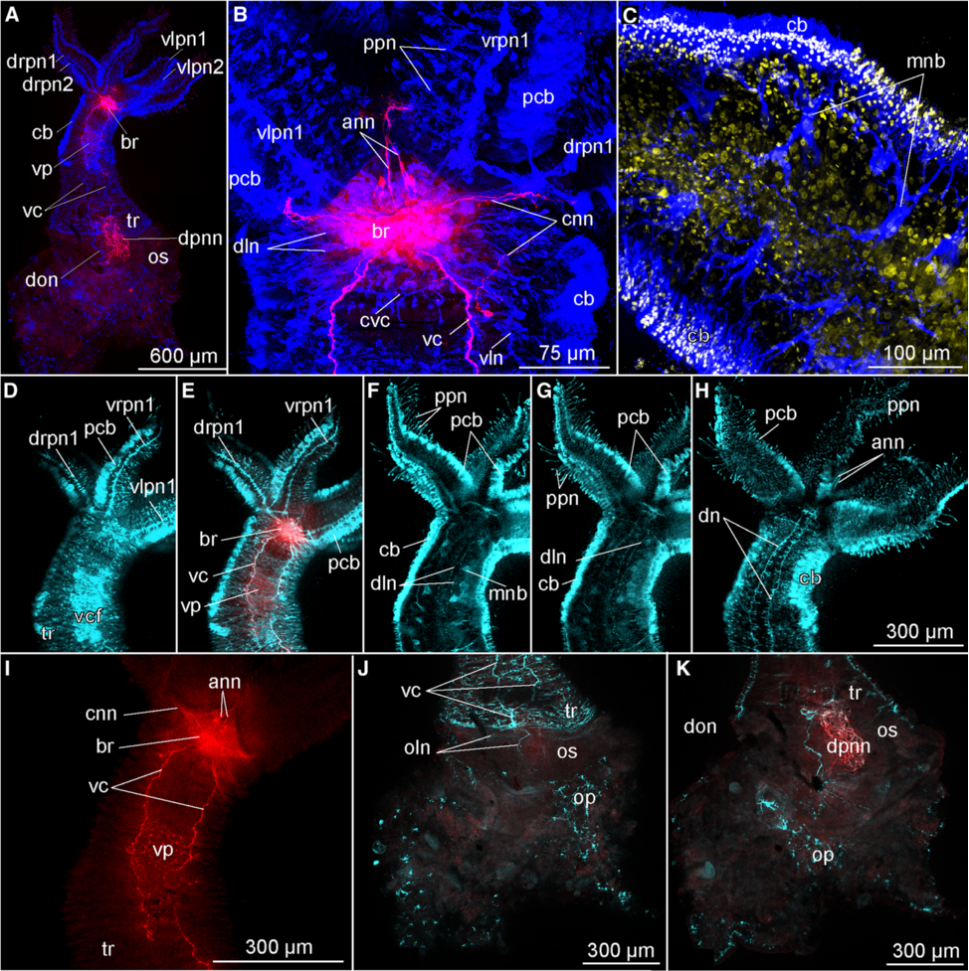
Confocal laser scanning microscopy of female *Osedax priapus*. **a** Overview, ventral view (red, serotonin LIR; blue, acetylated α-tubulin IR). **b** Anterior part with palps bases, ventral view (red, serotonin LIR; blue, acetylated α-tubulin IR). **c** Middle part of trunk (blue, acetylated α-tubulin IR; yellow and almost white, dapi). **d**-**h** Series of anterior region from the ventralmost side in (**d**) to dorsalmost side in (**h**) (red, acetylated serotonin LIR; cyan, acetylated α-tubulin IR; scale bar is similar for these pictures). **i** Serotonin LIR in anterior region, ventral side. **j**, **k** The ventralmost and dorsalmost sides of the posterior trunk region and ovisac, respectively (red, serotonin LIR; cyan, acetylated α-tubulin IR). Abbreviations: *ann* – anterior nerve net, *br* – brain, *cb* – ciliary band, *cnn* – circular nerve net, *cvc* – commissure of the *vc*, *dn* – dorsal nerve, *dln* – dorso-lateral nerve bundle, *dpnn* – dorsal posterior nerve net, *drpn1, 2* – dorsal right palp nerve 1, 2, *mnb* – neurites possibly innervating longitudinal muscles (for fast retraction), *oln* – ovisac longitudinal nerves, *op –* ovisac nerve net, *os* – ovisac, *pcb* – palp ciliary band, *pnn* - posterior nerve net, *tr* – trunk, *vc* – ventral cord, *vcf* – ventral ciliary field, *vln* – ventro-lateral nerve, *vlpn1, 2* – ventral left palp nerve 1, 2, *vrpn1* - ventral right palp nerve 1, *vp* – ventral plexus between *vc*

**Fig. 8:**
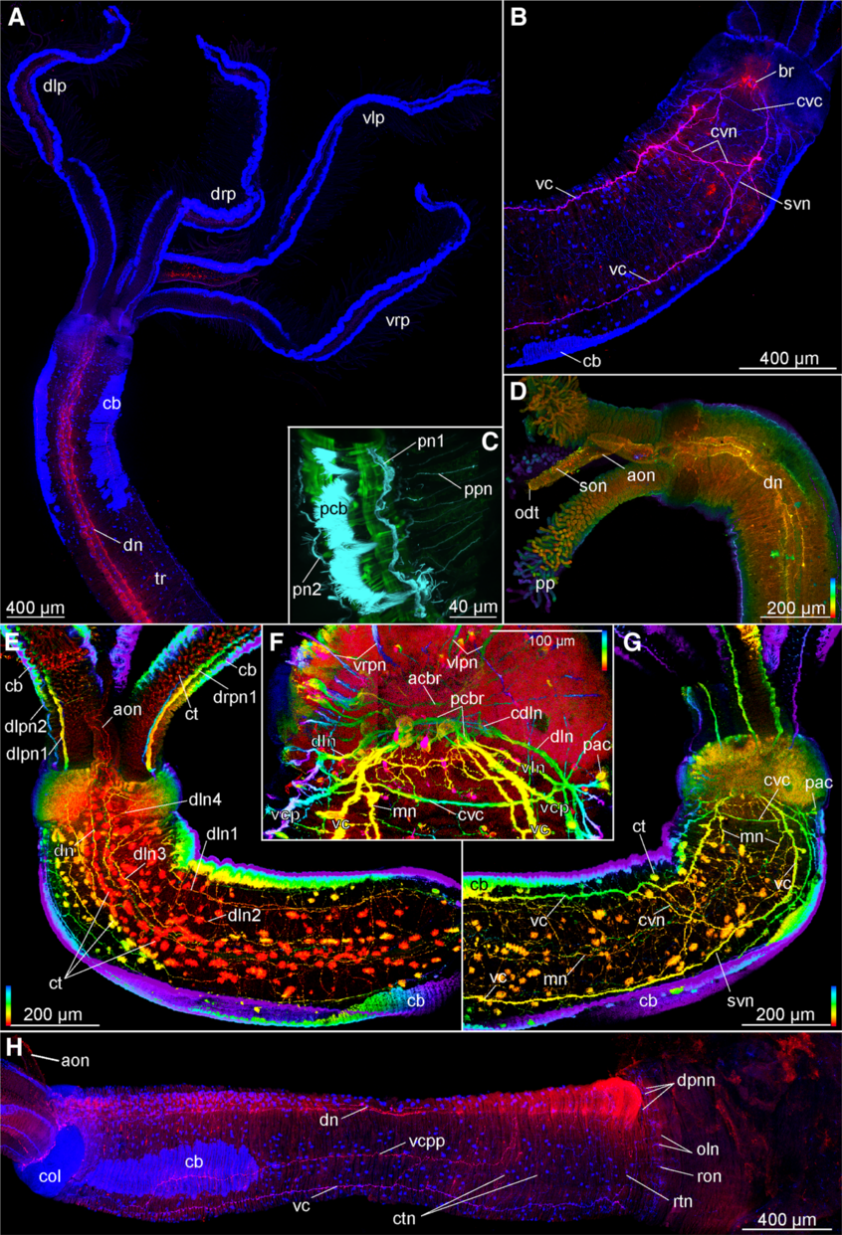
Confocal laser scanning microscopy of female *Osedax* “yellow collar”. Depth coded image (**g**) indicate the depth of the signal in the Z-stack: scale follows the colors of the spectral light, the top is to the surface. **a** Dorsal view (blue, acetylated α-tubulin IR; red, serotonin LIR). **b** Anterior end, ventral view (blue, acetylated α-tubulin IR; red, serotonin LIR). **c** Close up of a palp; muscles and nerves (green, phalloidin; cyan, acetylated α-tubulin IR). **d** Anterior end, dorsal view (anterior end at the left side; red, serotonin LIR). **e** Anterior end, dorsal view (depth coded colors, acetylated α-tubulin IR). **f** Cephalon and brain, ventral view (depth coded colors, acetylated α-tubulin IR). **g** Anterior end, ventral view (depth coded colors, acetylated α-tubulin IR). **h** Dorso-lateral view (anterior end at the left side; blue, acetylated α-tubulin IR; red, serotonin LIR). Abbreviations: *acbr* – anterior commissure of the brain, *aon* – anterior oviduct nerve, *br* – brain, *cb* – ciliary band,*cdln* – commissure of the dorso-lateral nerves, *col* – collar, *ctn* – circular trunk neurites, *ct* – ciliary tuft, *cvc* – commissure of the ventral cords, *cvn* – crossing neurites of the ventral nerve cord, *dn* – dorsal nerve, *dln* – dorso-lateral nerve bundle, *dln1-4* – dorso-lateral nerves 1-4, *dlp* – dorsal left palp, *dlpn1,2* – dorsal left palp nerve 1, 2, *dpnn* – dorsal posterior nerve net, *drp* – dorsal right palp, *drpn1* – dorsal right palp nerve 1, *mn* – median nerve, *odt* – oviduct tip, *oln* – ovisac longitudinal nerves, *pac* – perikarya of the *cvc*, *pcb* – palp ciliary band, *pcbr* – posterior commissure of the brain, *pn1,2* – palp nerve 1, 2, *pp* – palp pinules, *son* – nerves of sensory oviduct cilia, *svn* – straight ventral neurites of the *vc, tr* – trunk, *vc* – ventral cord, *vcp* – ventral cord projection, *vcpp* – posterior process of the *vc*, *vlp* – ventral left palp, *vlpn* – ventral left palp nerve, *vrpn* - ventral right palp nerve, *vrp* – ventral right palp, *vp* – ventral plexus between *vc*

**Fig. 9:**
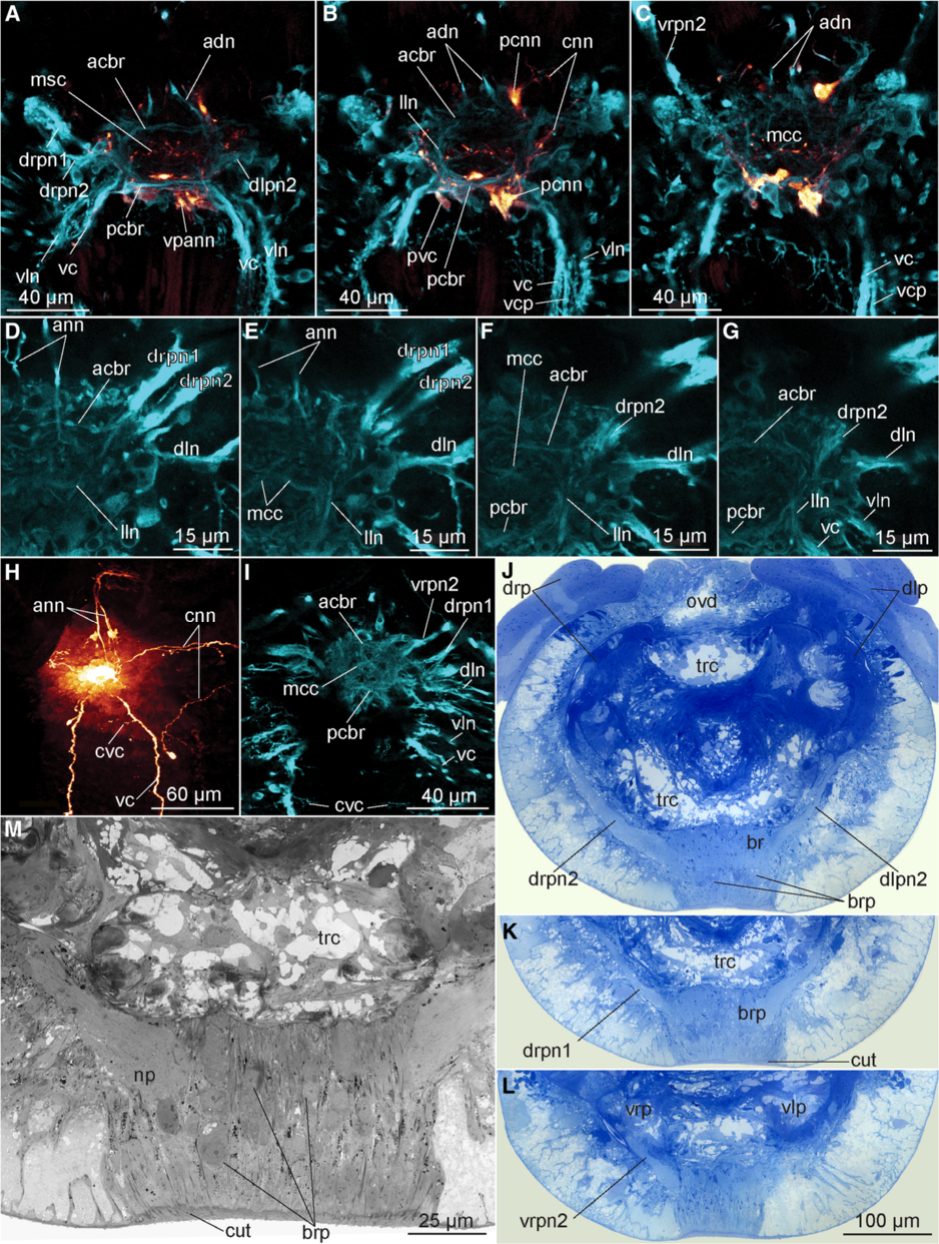
Brain organization of females of *Osedax* spp., Confocal laser scanning microscopy (CLSM) and light microscopy (LM). **a**-**c** Frontal CLSM optical sections of the brain of *O*. “nudepalp E” from the most dorsal in (**a**) to the most ventral part in (**c**) (cyan, acetylated α-tubulin IR; red, serotonin LIR). **d**-**g** Frontal CLSM optical sections of the right side of the brain of *O. mucofloris*, from the most ventral in (**d**) to the most dorsal side of the brain in (**g**) (cyan, acetylated α-tubulin IR). **h** Brain and associated neural elements of *O. priapus* (red, serotonin LIR). **i** Frontal CLSM optical section through the median part of *O. priapus* (cyan, acetylated α-tubulin IR). **g**-**l**. Transverse histological sections of the anterior trunk in the area of the brain, from the middle part of the brain in (**j**) to the anterior part of the brain in (**l**). **m** LM precise view of the anterior part of the brain showing the perikarya concentration. Abbreviations: *acbr* – anterior commissure of the brain, *adn* – antero-dorsal nerve, *ann* – anterior nerve net, *br* – brain, *brp* – brain perikarya, *cnn* – circular nerve net, *col* – collar, *cut* – cuticular shield, *cvc* – commissure of the ventral cords, *dln* – dorso-lateral nerve bundle, *dlp* – dorsal left palp, *dlpn2* – dorsal left palp nerve 2, *drpn1, 2* – dorsal right palp nerve 1, 2*, lln* – lateral longitudinal neurite bundles in the brain, *mcc* – middle cross commissure, *msc* – middle serotonergic commissure, *ovd* – oviduct, *pcbr* – posterior commissure of the brain, *pcnn* – perikarya of the cnn, *pvc* – perikarya of the vcrn – root nerve, *trc* – trunk coelom, *vc* – ventral cord, *vcp* – ventral cord projection, *vln* – ventro-lateral nerve, *vlp* – ventral left palp, *vpann* – ventral perikarya of ann, *vrpn 2* - ventral right palp nerve 2, *vrp* – ventral right palp

The female neuoarchitecture is highly similar among the four examined species of *Osedax* (Figs. 1, 2, 3, 4, 5, 6, 7, 8 and 9), whereas the large-sized *O. priapus* male deviates slightly, following its anatomical differences [29] (Fig. 3). Therefore, the common nervous system is described, naming the few interspecific differences within the description of each part. Most of the nervous system seemingly shows immunoreactivity (IR) to anti-acetylated α-tubulin (acetylated α-tubulin IR), whereas only subparts of the nervous system show immunoreactivity to the neurotransmitter stainings, anti-serotonin (serotonin-like (L) IR) and anti FMRF-amide (FMRF-amide like(L) IR) (Figs. 1 and 3).

The female nervous system is composed of an anterior, ventral brain (cerebral ganglion) from which originates the following main longitudinal nerves: four pairs of nerves entering the palps, two pairs of ventral nerves, two pairs of dorsal nerves, a pair of anterior oviduct nerves, and a pair of dorso-lateral nerve bundles. The two widely separated ventral cords are connected by a single distinct commissure in the anterior trunk and possibly by an additional commissure in the posteriormost trunk (or nerve net, see below). From the ventral cords project a range of smaller longitudinal nerves and plexi. A sparse peripheral nerve net, which originates from the ventral and dorsal cords, spans the trunk laterally and surrounds the posterior ovisac and root system. With the lack of an intestinal system, no stomatogastric nervous system or remnant thereof were found. The *O. priapus* male nervous system resembles this configuration, except for having i) two sets of palp nerves for its two palps, ii) anterior nerves not projecting into oviduct (lacking), iii) fewer longitudinal projections from the main longitudinal nerves.

### Brain

#### *Visualized with acetylated α-tubulin IR, serotonin LIR, and FMRFamide LIR* as well as histology and TEM

The ventral brain is intraepidermal and consist of a large neuropil, which ventrally and laterally is covered by densely packed perikarya located immediately next to the neuropil; visible with anti acetylated α-tubulin stain in a few specimens (Figs. 2, 5c and 6f).

Only 6-14 perikarya of the multiple brain perikarya were showing serotonin LIR (14 in *O.* “nudepalp E”, 8 in *O. priapus* female (10 in male), and 6 in *O.* “yellow collar”) and an indistinct number of more than 10 perikarya were showing FMRFamide LIR in *O. mucofloris* Glover, Källström, Smith, Dahlgren, 2005 [25] (not all shown; Figs. 2b, 3, 4a, b, 6d, 8b and 9h).

The neuropil is traversed by two main commissural bundles (anterior and posterior (acbr, pcbr, Fig. 2)) as well as by several minor transverse and decussating neurites (mcc, Fig. 2). It is not possible to follow the individual neurites of the dense neuropil with the data acquired. An attempt to trace the origin within the brain of the main longitudinal nerves is given in the description of each nerve group below. However, future detailed studies including histology are necessary in order to unravel a more exact number of commissures, their detailed configuration, and the precise origin of longitudinal nerves within the brain.

### Palp nerves

#### Visualized with acetylated α-tubulin IR and in parts by FMRFamide LIR

Each of the four palps (only two in male *O. priapus*) is innervated by two dense longitudinal nerve bundles, extending from the dorsolateral side of the neuropil of the anterior brain (Fig. 9j) to the distalmost end of the palps (pn1-2, Figs. 1, 2, 4a, b, 5a-e, 6a-b, e-f, 7b, 8a, d-f, 9a-g, i-m). Each dense palp nerve bundle originates as three discrete minor bundles from the anterior brain commissure, the posterior brain commissure and the lateral neuropil, respectively (Fig. 2). The separate origins within the brain suggest the presence of multiple neural functions such as motorneural control of palp musculature and ciliary beating as well as sensing of environmental cues. The three sets of nerves originating in the brain are gathered into two main bundles, lining each side of the palp. One of these nerve bundles (pn1, Fig. 8c) extends along the abfrontal palp side, from which minor transverse nerves project around the palp to the frontal surface, where numerous perpendicularly positioned pinnules are located. In each of these pinnules, a sensory neuron with a distally positioned perikaryon runs from the externally ciliated distal tip (ct, Fig. 8e) and along the central core before seemingly connecting with the transverse palp nerves at the base of the pinnules (Figs. 4c-e and 5a in [58]; and in *O.*”yellow collar” Fig. 8c). A second palp nerve (pn2, Fig. 8c) runs laterally underneath the one lateral, metachronously beating, ciliary band (out of two), which in fact are continuous around the palp tip. Specific innervation of the ciliary band could not be traced, with the acetylated α-tubulin IR in the small nerves being indistinguishable from the IR of cilia (no serotonin- or FMRFamide LIR were found in these small nerves).

### Ventral nerves (and projections from these)

#### Visualized with acetylated α-tubulin IR, and in parts by serotonin LIR & FMRFamide LIR

**Fig. 10:**
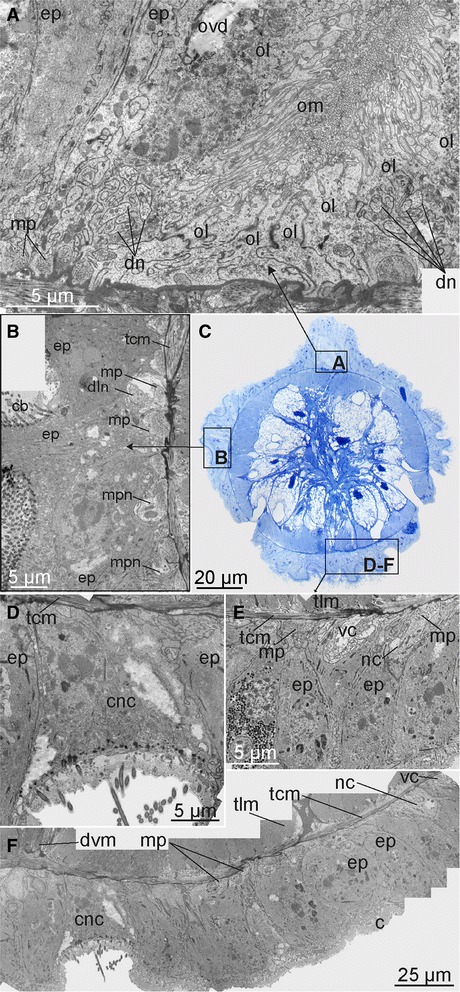
Intraepidermal elements of CNS in the trunk of female *Osedax priapus*. **a**, **b**, **d**-**f** TEM of ultrathin transverse sections. **c** Light micrograph of transverse histological section. **a** Dorsal fragment of section (**c**) showing the oviduct with dorsal neurites. **b** Lateral fragment of section (**c**) showing ciliary band and underlined dorsolateral neurites. **c** Transverse histological section of the trunk, frames indicate the fragments **a, b, d-f** with TEM views. **d-f** Neural elements of the epidermis of the ventral side: **d** Submerged ciliary cell at the middle of the ventral side. **e** Right bundle of the ventral cord and neural process next to it. **f** Overview of the ventral neural elements. Abbreviations: *c* – cuticle, *cb* – ciliary band, *cnc* – ciliary neural cell, *dn* – dorsal nerve, *dln* – dorso-lateral nerve bundle, *dvm* – dorso-venral mesenterium, *ep* – epidermal cells, *ovd* – oviduct, *mp* – muscle processes surrounded by the membrane and stretching at the bases of epidermal cells, *mpn* – cellular process with actin fibers and cytoplasm with granules, *nc* – neural cells, *ol* – epidermal cells lining the oviduct, *om* – microvilli projecting to the oviduct lumen, *tcm* – trunk circular muscles, *tlm* – trunk longitudinal muscles, *vc* – ventral cord

Widely separated paired main ventral nerves (here named ventral cords; vc, Figs. 1, 4a-e, 5a, c-e, 6c, d, f, 7a, e, i, j, 8b, f, g, h and 10e-f) originate from the anterior and posterior commissures within the brain (acbr, pcbr, Figs. 2, 9a, b, d, e, g and 10) and project from the ventro-posterior border of the brain, continuing posteriorly along the ventral side of the trunk, to the ovisac. An additional shorter pair of ventro-lateral longitudinal nerves (vln, Fig. 2) originates at the posterior brain commissure and extend postero-laterally underneath the lateral ciliary bands of the trunk to the posterior trunk, where they terminate (vln, Figs. 1a, c, 2, 3, 4a-c, 5a, c-e, 6f, 7b, 8f and 9a, b, g, i). The main ventral cords are interconnected by the distinct semicircular anterior trunk commissure (cvc, Figs. 1, 3, 4a, 5c, 7b, 8b, f, g and 9h, i) and possibly by the less distinct posterior trunk commissure, represented by a loose commissural nerve net forming a ring of nerves (rtn, Figs. 1, 4d-f and 8h).

The main nerve bundles of the ventral cords continue straight along the trunk (svn, Figs. 1a, b, 4d, i and 8b, g), with a few neurites decussating to meet the contralateral cord one-third down the trunk length (cvn, Figs. 1a, b, 4d, i and 8b, g, not shown for *O.* “nudepalp E” and not distinguishable in *O. priapus*). A midventral ciliary nerve plexus between the ventral cords (possibly disguising the decussating neurites) is found in *O. priapus* underneath its midventral ciliary field (vp, vcf, Fig. 7d, e, i); neither of which are present in any of the other examined species. Some minor neurite bundles project shortly from the main ventral bundles (not in female *O. priapus*), all terminating in the middle part of the trunk: i) one pair projects latero-posteriorly, anterior of the anterior trunk commissure (vcp, Figs. 1a, b, 2, 3, 4a, 5a, c, d, 6f, 8f and 9c); ii) one pair projects medially, posterior of the anterior trunk commissure (mn, Figs. 1a, 4a, b, 5d and 8f, g); iii) further posterior, one or two pairs project latero-posteriorly and meet dorsally in the middle trunk (vcpp, Figs. 1a, b and 8h). In the posteriormost trunk, the main ventral cords approach the contralateral nerve midventrally, forming a loose commissural nerve net (vpnn, Fig. 1a and 4e), from which project numerous transverse neurites, encircling the posterior trunk (rtn, Figs. 1, 4d, e, f, 6a and 8h). From this loose bundle of circular nerves (intermixing with circular peripheral nerves found densely distributed along the entire trunk) several short longitudinal nerves extend posteriorly (oln, Figs. 1a, c, 4a, e, f, 6a, 7j and 8h), possibly innervating the longitudinal muscles likewise condensed into bundles in the posteriormost trunk (Fig. 4a, in [57]). Several peripheral nerves encircle the trunk at the border to the ovisac (ron, Figs. 1a, c, 4a and 8h), through which the small longitudinal nerves (oln) extend across the anterior ovisac; together these nerves create a loose peripheral posterior nerve net, spanning the ovisac and roots (Figs. 1a, c, 4a, e, f, 6a, 7j and 8h).

Whereas all ventral nerves and commissures show acetylated α-tubulin IR, only the main ventro-lateral longitudinal nerves and the main bundles of the ventral cords as well as the posterior trunk ring nerve show FMRFamide LIR (ron, vc, vln, Figs. 1c and 4a-d). The main ventral nerve bundles and most of their projections showed serotonin LIR (cvn, rtn, vcp, vcpp, Figs. 1b, 3, 5d, e, 6a-c, 7a, b, i, k and 8a, b, h), but no signal was found in the ventro-lateral nerves or the circular commissural trunk nerves (rtn).

### Dorsal nerves

#### Visualized with acetylated α-tubulin IR, and in parts by serotonin LIR & FMRFamide LIR

A pair of dorsal nerve bundles originates from the anterior and posterior ventral brain commissures (in *O*. “yellow collar” most processes are from the posterior brain commissures) and extend antero-dorsally around the prostomial edge (adn, Figs. 1, 2, 5a-c, e, 6e, f and 9a-c). Dorsally, these nerve bundles split into three nerves: one short pair connecting with the anterior oviduct nerve; one pair running ipsilaterally across the anterior trunk and continuing straight along the entire dorso-lateral side of the trunk (ddn, only visualized with acetylated α-tubulin IR and not found in *O.* “yellow collar” and *O. priapus*; Figs. 1a, 5b and 6b, e); and one mid-dorsal pair continuing straight underneath the oviduct to the posterior trunk ring nerve (dn, showing IR against all nerve stains; Figs. 1, 3, 4a-d, g, h, 5b, d-f, 6a-c, e, 7h, 8a, b, d, e, h and 10a). The closely positioned mid-dorsal nerves are interconnected ventral of the oviduct by numerous transverse neurites, projecting from the chain of perikarya lining each dorsal nerve and showing bright serotonin LIR (Figs. 1, 3, 4g, h, 6a-c, 7h and 8a). In the anteriormost and posteriormost part of the dorsal nerve cords are found two dense serotonergic nerve plexi (ann and dpnn, Figs. 1a, 4f, g, h, 7k and 8h), possibly interconnecting with the loose circular nerves extending from the ventral side of the posteriormost trunk (rtn, Figs. 1b, 4d, g, h, 6a and 8h).

### Anterior oviduct nerves

#### Visualized with acetylated α-tubulin IR, serotonin LIR & FMRFamide LIR

On each lateral side of the brain, posterior of the eight palp nerves, a pair of anterior oviduct nerves (aon, Figs. 1a-b, 2, 4d, 5b, e, 6a, b, e, 8e, h and 10a) originates from the brain connectives (con, Fig. 2). They extend laterally around the anterior trunk, before they on the dorsal side make a 90 degrees turn in anterior direction, connect with the one projection of the antero-dorsal nerves, and continue along the free part of the anterior oviduct to its tip. At the tip the neurite bundles fan out with several of the highly serotonergic neurites connecting to externally ciliated sensory cells (Fig. 5b).

### Dorso-lateral nerves

#### Visualized with acetylated α-tubulin IR, and in parts by FMRFamide LIR

A conspicuous pair of dorso-lateral nerves (dln, Figs. 1a, c, 2, 3, 4a, c, 5a-e, 6e, f, 7g, 8e-g, 9d-g, i and 10b) originates at the posterior brain commissure (Figs. 2, 8f and 9f, g, i). Each dorso-lateral nerve bundle extends laterally from the brain (posterior of the oviduct nerves), and continues laterally around the anterior trunk, where after they split up into four-five dorso-lateral longitudinal nerves on each dorso-lateral side (dln1-5, Figs. 1a, c, 2, 3, 4a, 5a-e, 6e-f, 7b, g, 8e, f). The two ventralmost nerves underline the dorso-lateral ciliary band of the trunk (dln1-2), whereas the two-three dorsalmost nerves (dln3-5) all proceed around the lateral sides to meet up dorsally with the dorsal longitudinal nerves (dn) and continue alongside these dorso-posteriorly to the posteriormost trunk (Figs. 1a, c, 5b, e, 6e and 7g). In the smaller sized species *O. priapus,* only three dorso-lateral nerves could be traced (in both males and females) that all continue dorsally (Figs. 3 and 7b). The nerves are visualized only with acetylated α-tubulin IR, except for the two ventralmost ones, also seen with FMRFamide LIR.

## Discussion

### Comparison of nervous systems among female *Osedax spp.*

There are only slight differences in neural architecture among the various studied species, which all possess the main longitudinal nerve bundles (vc, vln, dln, dn) and the brain- and anterior trunk commisures (cvc). However, some of the minor projections and peripheral nerves could not be located in all species. In particular the crossing neurites of the main ventral nerves (cvn), the circular neurite bundles in posterior trunk (ctn, rtn), the median nerve (mn) and the posterior processes of the main ventral nerves (vcpp) could not be located in *O.* “nudepalp E” and *Osedax priapus. Osedax priapus* females further differ by the absence of ventral cord projections (though the male has these), and by the presence of a ventral plexus underlying the ventral ciliary field in the trunk (vp, vcf, Fig. 7d, e). Despite the nerve plexus (innervating the ciliary field) originates from the ventral cords, the overall similarity in numbers and configuration of ventral cords, however, indicates that the ventral and ventro-lateral longitudinal nerves themselves are not closely functionally linked with the absence/presence of a midventral ciliary field. This connection was otherwise suggested (but not shown) between the paramedian longitudinal nerves and the midventral ciliary band in some interstitial annelids [40, 60].

### Comparison of nervous systems among female *Osedax*, bone-eating males of *O. priapus* and dwarf males of *Osedax* spp.

Contrary to the commonly found microscopic dwarf males, the bone-eating male *O. priapus* is somewhat similar in size and anatomy to the females, though showing only two palps (not four), a spermioduct instead of an oviduct and a testis sac instead of an ovisac. The male nervous system in the head and trunk of *O. priapus* is likewise very similar to females, except for males having only four neurite bundles all originating at the dorsal neuropil of the anterior brain (Fig. 3) rather than having eight bundles originating along both the dorsal and lateral sides of the anterior brain (Figs. 7b, e and 9i). Furthermore, the seminal vesicle is innervated via anterior seminal neurite bundles extending from the median paired serotonergic perikarya in the brain (asn, Fig. 3) and forming dense nerve net in the wall of the seminal vesicle (snn, Fig. 3) possibly homologous to the anterior oviduct nerves found in females.

**Fig. 11:**
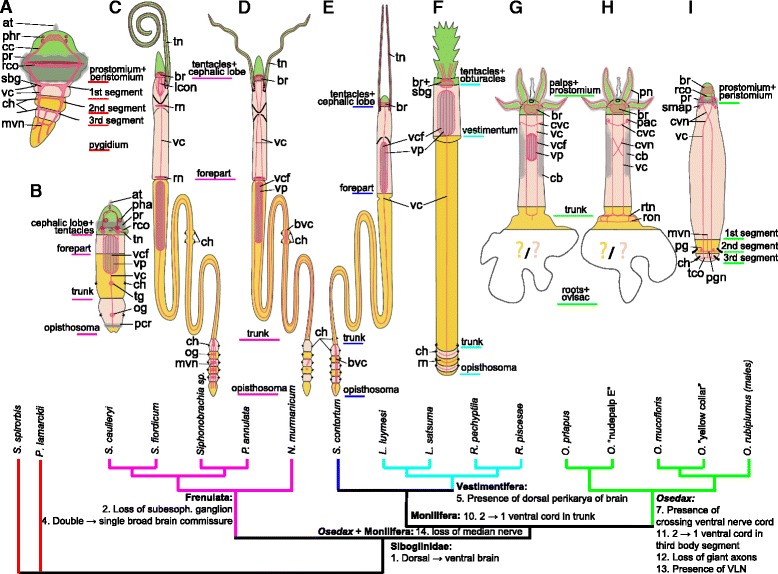
Evolution of nervous system and homology of segments and body regions in Siboglinidae. Consensus phylogenetic tree constructed from a combination of recent molecular phylogenies: clade of *Osedax* spp. resolved based on [29], Vestimentifera - [27], Frenulata - [1, 16], interrelationship of these clades - [27], serpulid outgroups - [13, 18, 19]. Neural characters provided in Table 1 are traced on the tree showing the 10 unambiguous character transformations on the main branches (only apomorphic states shown; for plesiomorphic states see Table 1). **a**-**i** Schemes of the main ventral cords and brains in Siboglinidae. Green color indicate fused prostomial + peristomial region; pink and yellow colors indicate following body segments of suggested homology among siboglinids and the metatrochophore of *Pomatoceros lamarckii*. Nerves and perikarya are drawn in red; ciliary bands in grey. Sidebars indicate body regions according to traditional siboglinid terminology. **a** Metatrochophore of *P. lamarckii* (Serpulidae)[61]. **b** Metatrochophore of *Siboglinum poseidoni* (Frenulata) [68]. **c** Combination of adult *Siboglinum caulleryi* (most of the body [64]) and *S. fiordicum* (opisthosoma)(Frenulata)[53]. **d** Adult *Nereilinum murmanicum* (Frenulata)[55]. **e** Adult *Sclerolinum contortum* [26]. **f** Juvenile *Riftia pachyptila* (Vestimentifera)[45]. **g** Female *Osedax priapus*. **h** Female *O. mucofloris*. **i**. Dwarf male of *O. rubiplumus* [12]. Abbreviations: *at* – apical tuft, i – brain, i – broadened ventral cord, *cc* – circumesophageal connectives, *cb* – ciliary band, *ch* – chaetae, *cvc* –commissure of *vc*, *cvn* – crossing neurites of *vc*, *mn* – median nerve, *mvn* - median ventral nerve, *og* – opisthosomal ganglion, *pcr* – telotroch, *pg* – posterior ganglion, *pgn* – posterior ganglion nerve, *pha* – phaosome, *phr* – photoreceptor, *pn* – palp nerve, *pr* – prototroch, *pac* – perikarya of *cvc*, *rco* – prototroch ring commissure, *rn* – ring nerve, *ron* – ring ovisac nerve, *rtn* – posterior trunk ring nerve, *sbg* – subesophageal ganglion, *smap* – serotonergic perikarya of *vc*, *tco* – terminal commissure, *tn* – palp nerve, *tg* – trunk ganglion, *vc* – ventral cord, *vcf* – ventral ciliary field, *vp* – ventral plexus between *vc*

The dwarf male nervous system was previously described by immunocytochemistry and CLSM [12] and despite the great size difference of the microscopic males, their nervous system seemingly shows several homologous traits with that of bone-eating *Osedax* forms: For instance, the dwarf male nervous system comprises an anterior ventral brain with two commissures, connected to paired ventral, ventro-lateral, dorso-lateral and dorsal longitudinal nerves (though the dorsal nerves could not be traced along the mid-trunk). Besides the similar number of main cords, the most striking resemblance is the anterior decussating neurites of the ventral cord (Figs. 1, 11, fig. 6 in [12]). The homology of the transverse commissures is less easy to depict, but the first posterior trunk commissure may resemble the posterior trunk commissure in females (Figs. 1, 11, fig. 6 in [12]). The anterior trunk commissure (cvc) found in all females and *O. priapus* males, was not found in dwarf males. With the fewer neurites generally found in dwarf males it may be overlooked, or lacking. However, there is an anterior nerve ring beneath the anterior ciliary bands (prototroch). We do not consider this homologous to the more posteriorly located cvc commissure of females, since the dwarf male nerve ring seems closely related to their prototroch ciliary patches. Moreover, similar prototroch nerve rings with similarly associated posterior perikarya are found in CLSM studies of other annelid larvae [61, 62].

### Comparison with nervous systems of other Siboglinidae

The neuromorphology of *Osedax* shows overall resemblance to other siboglinids in having an intraepidermal, ventral brain and anteriorly unsegmented nerve cord with few commissures and plexi along an elongated trunk (Table 1 and references herein). However, it also seemingly differs from other siboglinids in having additional ventrolateral and dorsolateral longitudinal nerves (Table 1 and references herein), as well as having multiple longitudinally running projections from all the main nerves, together creating a relatively dense fence of longitudinal nerves surrounding the trunk. This fence-like configuration may accomodate the innervation of the surrounding sheath of longitudinal muscles as well as the broad longitudinal ciliary bands of the trunk. Other siboglinids seemingly lack these paired lateral ciliary bands, possibly explaining their fewer lateral nerves (Table 1 and references herein). However, even though these nerves are quite prominent in the small-sized *Osedax* spp., they may have been overlooked in the histological studies of larger sized siboglinids, where such details may be missed. The wide separation of the ventral cords along the entire trunk in *Osedax* differs from the general adult and larval configuration in Siboglinidae, where the ventral cords mostly fuse in the trunk midline (Fig. 11, Table 1 and references herein) and contain giant axons (lacking in *Osedax*). A midventral ciliary field is present in the anterior region of most larvae (= neurotroch) and adult siboglinids (albeit with somewhat different configurations; Fig. 11) and underneath this field the ventral nerve cords are always widely separated and interconnected by a nerve plexus (see Table 1 and references herein). A presumably homologous midventral ciliary field was found in female *Osedax priapus* (Fig. 7a, e, i), which was similarly innervated by a dense serotonergic nerve plexus interconnecting the widely separated ventral cords.

**Table 1:**
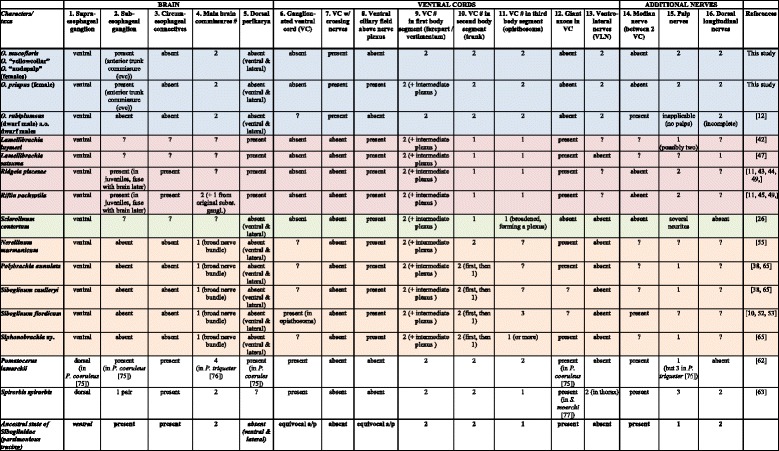
Main characteristics of the siboglinid nervous system [10–12, 26, 38, 42–45, 47, 49, 52, 53, 55, 62, 63, 65, 68, 75–77

Sixteen character are scored for the siboglinid species for which relatively detailed morphological studies exist. Blue codes features of *Osedax*, pink - Vestimentifera, green – *Sclerolinum*, brown – Frenulata. *Osedax mucofloris*, *O.* “yellow collar”, and *O.* “nudepalp E” had similar scorings and were therefore included in one common row. Investigated *Osedax* dwarf males likewise showed similar scores. References are given in the last column. If no information were available for the distinct species, data from closely related species were occasionally provided (with references). The evolution of the 16 characters was traced on the tree shown in Fig. 11 (using MacClade 4.08.a), which only displays unambiguously traced changes. The ancestral states for Siboglinidae of all 16 characters were likewise traced and provided in last row of the table; states written in italics being apomorphic (vs. plesiomorphic) of Siboglinidae

The present study shows that the two longitudinal nerves of each *Osedax* palp arise from the anterior- and, to a lesser degree, the posterior brain commissures. With the uncertain phylogenetic position of Siboglinidae, and the lack of mouth, gut and accordingly circumesophageal connectives, it is not possible to homologize the brain commissures in *Osedax* with the dorsal and ventral commissures found in most other annelids [40]. Two longitudinal nerves have been found in each of the so-called tentacles of some vestimentiferan *Riftia*, *Ridgeia* and *Lamellibrachia luymesi* van der Land and Nørrevang, 1975 [11, 42, 63], supporting their homology to *Osedax* palps, though their innervation has not been traced to the brain commissures. In the frenulates studied to date, only one nerve has been found in each also so-called tentacle [38, 64], underlining their presumably more distant relationship to *Osedax*, though presumably still being homologous to annelid palps *sensu* Orrhage and Müller [40].

*Osedax* specimens have two main (and several minor) brain commissures and one anterior trunk commissure arguably homologous to the supra- and subesophageal ganglia, respectively, found in most annelids. The vestimentiferan *Riftia* likewise show two main brain commissures, which fuse with the single commissure of the subesophageal ganglion during development [45, 48]. Frenulates have only one broad brain commissure and lack a subesophageal ganglion in both larval and adult stages. Further developmental and detailed histological studies are warranted to fully address the commissural configuration and homology across Siboglinidae as well as annelids in general. With a better understanding of the brain commisures, the exact origin and homology of palps and longitudinal nerves can be assessed.

### Evolution of the siboglinid nervous system

The most parsimonious reconstructions of the neuromorphological characters (listed in Table 1) were traced (in MacClade 4.08a) on a consensus phylogeny for Siboglinidae, based on various phylogenetic results (*Osedax* [29], Vestimentifera [27], Frenulata [1, 16], interrelationship of these clades [27], and other Sedentaria that served as outgroups [13, 18, 19] (Fig. 11). Since *Osedax* only has a few segments, we found it most relevant to compare the details with those of late larval stages in outgroup taxa, when they have similarly few segments. Furthermore, the metatrochophore (in comparison to adults) has all basic body parts of annelids: prostomium + peristomium + larval segments + pygidium, whereas in adults of e.g., Sabellida the prostomium and peristomium fuse [13, 65]. For both of the two serpulid terminals shown in Fig. 11, the nervous system has been studied using comparable immohistochemical methods [61, 62].

Only unambiguous transformations (in 10 of the 16 characters) along the main siboglinid branches are discussed here (see Fig. 11). Only one unambiguous transformation supports Siboglinidae: character 1, the shift from dorsal to ventral position of the brain. The possible loss of a ganglionated cord (character 6) within Siboglinidae could not be traced unambiguously, since many siboglinids have not been investigated in such details to assess presence or absence (Table 1). Also, ganglionated cords are not consistently found in annelids [41], so the character transformation is further dependent on the choice of outgroup, which is as yet unresolved for Siboglinidae. The intraepidermal nervous system with widely separated nerve cords, double brain commissures, and double palp nerves and numerous other traits found in *Osedax* were all traced as plesiomorphic states for Siboglinidae, though not consistently present in all siboglinids (see Table 1, last row). Two unambiguous transformations characterize Frenulata: character 2, loss of subesophageal ganglion, and character 4, fusion of two brain commissures into one broad single commissure. Only one unambiguous transformation respectively characterized the *Osedax* + Monilifera clade (character 14, loss of median ventral nerve), Monilifera itself (character 10, fusion of widely separate cords into a single cord in the second body segment), and Vestimentifera (character 5, formation of a massive dorsal layer of the perikarya in the brain). For *Osedax* we traced four unambiguous transformations: character 7, decussating neurites of the ventral nerve cords; character 11, fusion of the two ventral cords into one in the third body segment; character 12, loss of giant axons; character 13, formation of multiple ventro-lateral nerve processes in the trunk.

With the few apomorphic states supporting the main clades, it is clear that a neurophylogeny would not alone be able to resolve the interrelationships of Siboglinidae. Also, the reconstruction depends on the topology and outgroups of the phylogenetic tree on which the characters are mapped, some of which are still debated. However, this was the first attempt within Annelida to assess neurophylogenetic characters and states. Several of these could be reconstructed unambiguously, thus providing significant information on transformations of the nervous system within Siboglinidae.

### Definition of segment borders and regions across different species and sexes of *Osedax*

Based on the presence of one pair of ganglia or one major commissure per segment, the general configuration of the nervous system, and the four pairs of chaetae found in dwarf males, we argue for the following body regionalization and up to three body segments being present in *Osedax* (Fig. 11): i) Fused prostomium and peristomium containing the brain with two main commissures, prototroch nerve ring underlying the prototroch (in larvae and dwarf males [12]), and palps innervated by nerves originating at the brain commissures (in females and male *O. priapus*); ii) First body segment containing the anterior trunk with an anterior commissure of the ventral cord (in females), and posterior to this, either a characteristic crossover of the ventral cords (found in all dwarf males [12] and most *Osedax* females or in *O. priapus* female a ventral nerve net underlying a ventral ciliary field; iii) Second body segment, which in the larval/dwarf male stage comprises a first set of chaetae as well as a ganglion, and in the females comprises a posterior trunk commissure (no chaetae) as well as possibly the ovisac and roots; iiii) Third trunk segment found in dwarf males containing a second set of chaetae and a terminal commissure (plus a telotroch posteriorly), which may resemble the first opisthosomal segment in other siboglinids. This contrasts with our previous intepretation of *Osedax* dwarf males as having only a total of two segments [12]. In females no opisthosomal or trunk chaetae have been found to date, and their posterior ovisac and roots may resemble either a posterior part of the second trunk segment in dwarf males or a third segment, though with no clear indication of segment borders (no ganglia and no dissepiments found in this part).

### Homology of body regions and segmentation in Siboglinidae

The prostomium and peristomium is not easily distinguished from the body segments in adult Siboglinidae, and the exact homology is not obvious. In *Osedax* we suggest that the anteriormost part of the body represents the fused prostomium + peristomium, again corresponding to the cephalic lobe seen in Frenulata and *Sclerolinum* (in accordance with [2, 26, 31], as well as the anteriormost part of the vestimentum in Vestimentifera [3]. The latter statement agrees with Rouse, 2001 [1] and with Bright et al. 2013 [31], which shows that they at least contain this region during development. All groups carry palps (sometimes referred to as tentacles) and a ventral brain in this region (Fig. 11a-i). A posterior demarcation of this region (prostomium + peristomium) is externally indistinguishable in all Siboglinidae, lacking a constriction, though a mouth opening may be present in larval stages. The external morphology of this region is relatively simple in *Osedax* larvae, only showing an apical tuft and a prototroch [66], whereas Vestimentifera and Frenulata larvae also show a mouth opening [31, 33, 67, 68], and frenulate larvae and adults possess phaosomes [68, 69]. Internally, the larvae (and dwarf males of *Osedax*) contain a prototrochal commissure (with associated paired dorsal and ventral ganglia found in Frenulata (Fig. 11b)), and all adult Siboglinidae possess a conspicious ventral brain ([12, 26, 31, 43, 49, 55, 64, 68], this study). In larvae of *Riftia* (Vestimentifera) this here suggested prostomial/peristomial region was shown to be posteriorly demarcated by the septa of the following first body segment, having a separate coelomic cavity [31]. Unfortunately, for all other Siboglinidae the coelomic cavities are either unstudied, less clearly interpreted, or as for Frenulata showing a different configuration [2, 31]. No cell lineage studies or gene expression studies have been performed of Siboglinidae, but studies of the annelids *Platynereis* [70, 71], *Hydroides* and *Capitella* [72] all confirm the presence of a proliferating stomodaeal region separate from the prostomium as well as the first body segment.

The first body segment develops posterior to the peristomium, and while the exact border between these two can be difficult to detect, we argue that the first segment of bone-eating *Osedax* is easily recognized by the presence of a pair of subesophageal ganglia/commissure, even though chaetae are missing [40, 60] (Fig. 11g-i). We further suggest that the first body segment in siboglinids is constituted by the forepart in Frenulata and *Sclerolinum* (following Rouse [1], but conflicting with Bright et al. [31], who claims that the first body segment also includes some of the trunk). The first segment is, moreover, equivalent to the middle part of the vestimentum in Vestimentifera (again in accordance with Rouse [1], but again disagreeing with Bright et al. [31], because they find the first segment to also include part of the trunk) [1, 26, 31], and lastly this is homologous to the middle part of the trunk in *Osedax* [56] (this study, Fig. 11g-i). Our proposed homology and demarcation of the first segment is supported by the present finding of an anterior commissure (cvc, =subesophageal ganglion) in the anterior trunk of bone-eating *Osedax*, as well as by the recovered ventral ciliary field with an underlying nerve net in *Osedax priapus*, similar to what has been reported for Vestimentifera, *Sclerolinum* and possibly the larvae of Frenulata [2, 26, 29, 68]. A presumed homologous subesophageal ganglion is found in Vestimentifera (fuses with the brain during develoment) and other annelids (e.g., [40, 45, 48, 61, 62] this study, Table 1, Fig. 11), though not reported for *Sclerolinum* [26], and in Frenulata may instead be represented by a ring nerve [55, 64].

The second body segment comprises the posterior part of the trunk in *Osedax,* demarcated by one pair of chaetae and a prominent commissure with ganglia in dwarf males, or by a posterior trunk ring nerve in the bone-eating forms (Fig. 11). In bone-eating forms this segment has previously been referred to as ‘trunk base’ or ‘lower trunk’ and it may extend into the ovisac (e.g., [29, 56]). We suggest it to be homologous to the trunk in Frenulata, *Sclerolinum* and Vestimentifera [2, 26], which in Vestimentifera has 1-2 pairs of chaetae (anterior capillary chaetae are lost during development) [12, 26, 55, 64, 68]. A homology to the *Osedax* dwarf male ganglia or posterior trunk commissure have not been found in other adult Siboglinidae, however, a single ganglion is present in frenulate larvae [68]. Our neuromorphological regionalization is further supported by other anatomical features such as presence of a second coelomic cavity delineated by dissipiments in larval stages of both Frenulata and Vestimentifera [31], as well as the presence of gonads and the trophosome in this segment in all adult Siboglinidae [2, 10, 11, 38, 43, 46]. However, this interpretation contradicts the regionalization suggested by Bright et al. [31] according to which, the trunk together with the forepart/vestimentum constitutes the first body segment and not a separate second segment.

The third body segment in most siboglinids equals the first segment of the opisthosomal region [2, 10, 11, 38], characterized by opisthosomal chaetae in all siboglinids except the bone-eating forms of *Osedax,* and is further characterized by a telotroch in larvae of *Osedax* and Frenulata [66, 68]. Developmental studies are necessary in order to clarify whether the ovisac and roots in bone-eating forms of *Osedax* likewise represent a third segment (or more) rather than a second segment, similar to the dwarf males. The hooked chaetae in *Osedax* dwarf males are all similar, whereas other siboglinids may show modified opisthosomal compared to trunk chaetae [12, 26, 55, 64, 68]. Neural architecture in the third segment comprises a terminal commissure in *Osedax* dwarf males [12], a pair of ganglia in Frenulata [53], broadening of the ventral nerve cord in *Sclerolinum* [26], or a neural circular bundle in Vestimentifera [45]. This organization is repeated in the following opisthosomal segments of other Siboglinidae, if present.

## Conclusions

The female *Osedax* nervous system revealed many similarities to the nervous system of other Siboglinidae but also differed in term of the presence of multiple, widely-separated longitudinal nerves, extending throughout the trunk, and by their lack of giant axons and a segmented opistosomal region. Moreover, the present, first, broader comparison of nervous system across *Osedax* and Siboglinidae has led to new proposed homologies of the ambiguous, and much debated, anterior segments in Siboglinidae - with *Osedax* females (and *O. priapus* males) suggested to comprise two in the trunk. *Osedax* dwarf males are here suggested to have three body segments in total (in contrast to the two interpreted as in [12]). This is also the first neurophylogenetic study performed on annelids and Siboglinidae, leaving several transformations unambigously reconstructed at the deep nodes. The reconstruction did reveal that the *Osedax* intraepidermal nervous system with multiple, widely separated nerve cords, double brain commissures, double palp nerves are siboglinid plesiomorphies; hereby bridging the evolutionary gap to non-siboglinid annelids and enhancing our understanding of the neural evolution within Siboglinidae.

## Material

For recruitment of female *Osedax mucofloris,* cow and whale bones were placed at 125 m depth off the coast of Tjärnö, Sweden, and successfully retrieved in 2009 [57], in close vicinity to a minke whale carcass sunk in October 2003 [73]. All necessary permits were obtained for the described whale fall experiment; received from Karin Pettersson at the County Administrative Board Västra Götalands Län, October 2003. The presently undescribed *Osedax* “yellow collar” was sampled in November 2009 in Monterey Bay, California (CA), the USA, at 385 m depth from a gray whale skeleton. *Osedax priapus* (males and females) were collected from bones of a northern fur seal and a northern elephant seal that were deployed in Monterey Canyon, CA, the USA, in 2009, and cow bones deployed at Hydrate Ridge, Oregon, in 2011 [29]. All bones were placed at 600-800 m depth. Collections of the undescribed *O.* “nudepalp E”, were made on specimens living on whale skeletons deployed at 683-1820 m depth in Monterey Bay, CA, the USA [66] in 2002-2006 [74]. *O.* “yellow collar” and *O.* “nudepalp E” are separate undescribed phylogenetic entities as revealed by a molecular phylogeny [37].

## Methods

### Fixation

Animals were carefully anesthetized with magnesium chloride (7 %) in seawater, ratio 1:1, and then dissected from the bone. Specimens for immunohistochemistry were fixed in fridge overnight in 4 % paraformaldehyde in phosphate-buffered saline (PBS) containing 5 % sucrose. Samples were rinsed 5-7 times and stored in PBS-buffer (with 5 % sucrose and 0.05 % NaN_3_). Specimens for histology and TEM were fixed in 3 % glutaraldehyde in 0,1 M cacodylate buffer with 5 % sucrose and later postfixed in 1 % osmium for 1,5 hours.

### TEM, histology & LM photography

Prior to embedding in Spurr’s resin, specimens were dehydrated in alcohol series using standard protocol and thereafter polymerized for 20-24 hours at 50 °C. The block was trimmed to the object and sectioned into semithin (600-700 nm) and ultrathin (50-70 nm) sections using a Leica EM UC7 ultramicrotome (LEICA MICROSYSTEMS, Wetzlar, Germany). Semi-thin sections were stained with toluidine blue and photographed with an Olympus IX70 light microscope equipped with a digital Olympus camera DP73 using the program Cell (Olympus America Inc., Tokyo, Japan). Ultrathin sections were mounted on slot grids and mesh grids, contrasted with 2 % uranyl acetate- and 4 % lead citrate-solution and examined using a JEOL JEM 1010 and 1011 - Transmission Electron Microscopes (JEOL Ltd., Tokyo, Japan; belonging to University of Copenhagen and Papanin Institute for Biology of Inland Waters, Russian Academy of Science, respectively) equipped with a digital camera GATAN OneView (GATAN, INC., Pleasanton, CA, United States) and digital camera MEGA VIEW-III (Olympus America Inc., Tokyo, Japan), respectively.

### Immunohistochemistry

Immunohistochemical staining of muscles, nerves and cilia of two or more specimens of each species were performed synchronously as triple or double staining. The following antibodies and fluorochromes were employed for indirect and direct triple stainings: phalloidin labeled with FITC (0,17 μM and 0,33 μM stock solution in PBS; Sigma-Aldrich, St. Louis, MO, USA), or Alexa Fluor® 488 (0,33 μM stock solution in PBS; Invitrogen, Eugen, OR, USA); monoclonal mouse acetylated α-tubulin (final concentration 1:400; Sigma-Aldrich, T6793) (with CY5 labeled secondary antibody directed against mouse; Jackson Immuno-Research, West Grove, PA, USA); polyclonal rabbit anti serotonin (diluted 1:400; Sigma-Aldrich, S5545) and anti FMRFamide (diluted 1:100; Immunostar, Hudson, WI, USA, 20091) (both with TRITC-labeled secondary antibody directed against rabbit (Sigma-Aldrich, T5268)). The staining combinations (1:1) were acetylated α-tubulin/serotonin and acetylated α-tubulin/FMRFamide followed by direct phalloidin staining.

Samples were preincubated for at least 2 hours in PTA (PBS + 0.1 % Triton-X, 0.05 % NaN3, 0.25 % bovine serum albumin, and 10 % sucrose). Afterwards, samples were incubated for up to 24 hours at 4 °C in the primary antibodies mixed 1:1, both made with PTA. The samples were then thoroughly washed through several shifts with PTA over 6 hours and then incubated overnight at 4 °C in the two respective secondary antibodies conjugated with fluorochromes and mixed in PTA. The third day samples were again rinsed thoroughly over 4 hours with PTA and then incubated in phalloidin (in PBS without NaN_3_), either labeled with FITC (SIGMA P-5282) or Alexa flour 488 (Invitrogen A12379)(0,33 μM), for one hour. Samples were then rinsed 2-3 hours in PBS and before mounting taken through a series of increasing concentration of mounting media Vectashield® with DAPI (Vector Laboratories, Burlingame, CA, USA). Specimens were mounted on an object glass between two cover slips, so that they could be turned and scanned from both sides.

The same approach was used for double staining, except the direct phalloidin staining was excluded. The phalloidin staining was generally not shown for this study.

### Confocal laser scanning microscopy

Preparations were investigated with a Leica TCS SP5 Confocal Laser Scanning Microscope (SUND & SCIENCE, University of Copenhagen) performing Z-stacks of images (series of images through Z-plane of specimen stacked together), which were later carefully analyzed in order to reconstruct the nervous system using the Leica LASAF computer software and Imaris software (Bitplane, Concord, MA, USA), respectively. Maximum intensity and depth-coded 2D projections of Z-stacks were likewise constructed by help of these programs and later optimized for contrast and level in Adobe Photoshop CS 5.1 (Adobe Systems, San Jose, CA, USA). Drawings were performed with Adobe Illustrator CS6.
